# Vaginal mucosal necrosis following traditional vaginal cauterization in Somalia: a case report

**DOI:** 10.1093/jscr/rjag351

**Published:** 2026-05-06

**Authors:** Ikran Abdırahman Yusuf, Kadri Tezel, Yesim Kürekci, Hiba Bashır Hassan, Samira Ahmed Mohamud

**Affiliations:** Department of Obstetrics and Gynocology, Mogadishu Somali-Türkiye Recep Tayyip Erdoğan Training and Research Hospital, Digfer Street, Hodan District, Mogadishu, Somalia; Department of Obstetrics and Gynocology, Mogadishu Somali-Türkiye Recep Tayyip Erdoğan Training and Research Hospital, Digfer Street, Hodan District, Mogadishu, Somalia; Department of Infectious Diseases and Clinical Microbiology, Mogadishu Somali-Türkiye Recep Tayyip Erdoğan Training and Research Hospital, Digfer Street, Hodan District, Mogadishu, Somalia; Department of Obstetrics and Gynocology, Mogadishu Somali-Türkiye Recep Tayyip Erdoğan Training and Research Hospital, Digfer Street, Hodan District, Mogadishu, Somalia; Department of Obstetrics and Gynocology, Mogadishu Somali-Türkiye Recep Tayyip Erdoğan Training and Research Hospital, Digfer Street, Hodan District, Mogadishu, Somalia

**Keywords:** vaginal necrosis, thermal injury, traditional cauterization, pelvic organ prolapse, colpocleisis, global health

## Abstract

Traditional vaginal cauterization is practiced in some low-resource settings, including Somalia, for gynecological complaints. Unregulated thermal exposure may cause severe tissue destruction. Circumferential full-thickness vaginal necrosis requiring reconstruction is extremely rare. A 70-year-old multiparous Somali woman presented with severe vaginal pain, foul discharge, fever, and urinary retention 14 days after cauterization. Examination revealed stage IV pelvic organ prolapse and circumferential full-thickness vaginal necrosis extending to within 1 cm of the urethral meatus. Laboratory tests showed leukocytosis (12 400/mm^3^), elevated C-reactive protein (46 mg/L), and creatinine 1.3 mg/dL. Intravenous antibiotics were initiated. Wound culture grew *Klebsiella* spp. susceptible to gentamicin, and therapy was adjusted. Surgical debridement followed by colpocleisis was performed. Recovery was uneventful. Traditional vaginal cauterization may cause devastating thermal injury requiring radical surgery. Early recognition and multidisciplinary management are essential. Preventive public health measures are urgently needed.

## Introduction

Harmful traditional practices affecting women’s health remain a significant public health concern in low-resource regions. The World Health Organization has repeatedly emphasized that such practices may result in long-term gynecological morbidity and reproductive complications [1].

In Somalia, traditional vaginal cauterization involves direct application of heated metallic instruments to the vaginal mucosa, often performed without temperature regulation or sterile technique. Thermal injury leads to protein denaturation, microvascular thrombosis, tissue ischemia, secondary bacterial colonization, and progressive full-thickness necrosis.

Although superficial genital burns and infections have been described, circumferential full-thickness vaginal necrosis requiring obliterative reconstructive surgery has not been clearly documented.

## Case presentation

### Clinical findings

A 70-year-old, para 8 Somali woman presented with severe vaginal pain, malodorous purulent discharge, fever (38.5°C), acute urinary retention. Symptoms developed 14 days after undergoing traditional vaginal cauterization. Pelvic examination revealed stage IV pelvic organ prolapse (POP-Q), circumferential yellow necrotic tissue involving the entire vaginal wall, necrosis extending to 1 cm below the urethral meatus, copious purulent discharge.

**Figure 1:**
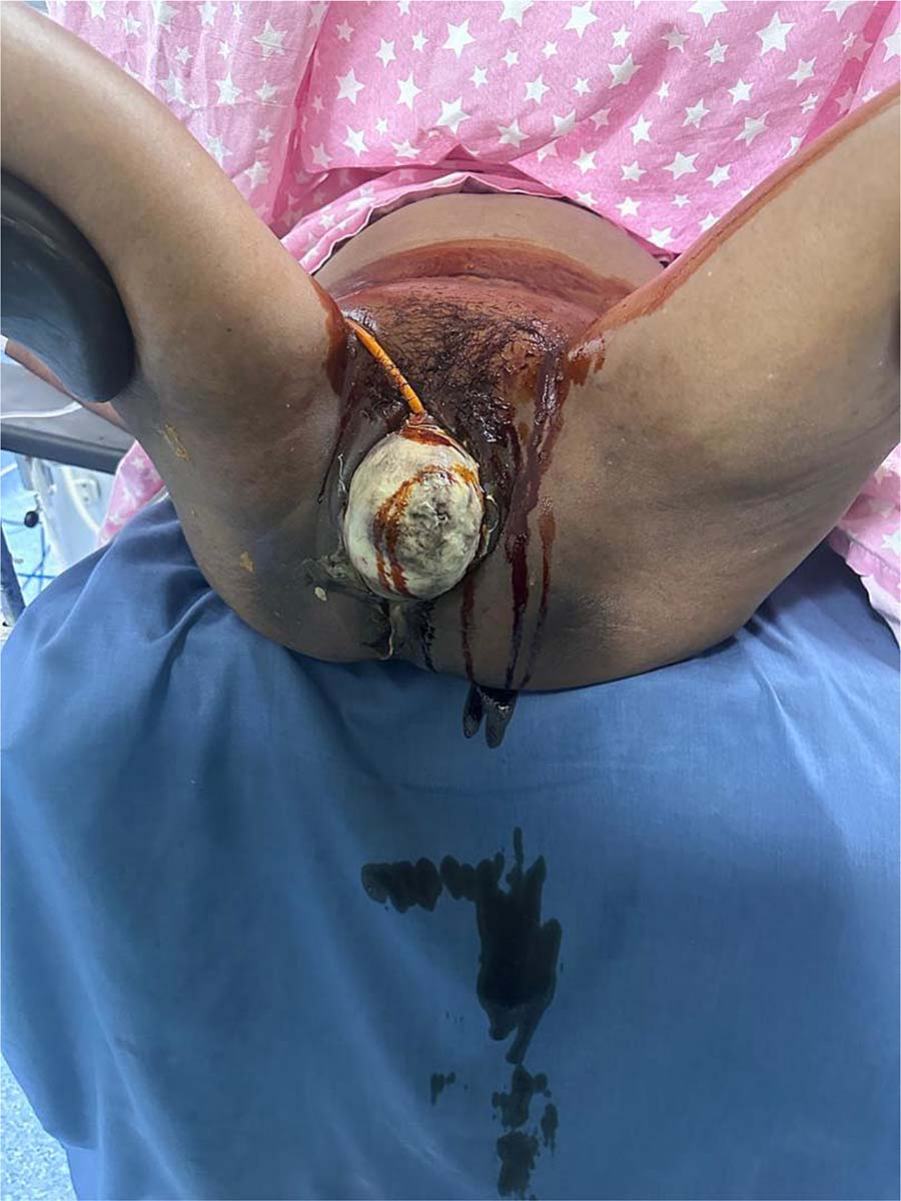
Preoperative image demonstrating stage IV pelvic organ prolapse with circumferential full-thickness vaginal necrosis secondary to thermal injury.

The full-thickness necrotic destruction of the prolapsed vaginal wall is shown in Fig. 1.

### Diagnostic assessment

Laboratory investigations Leukocyte count 12 400/mm^3^, CRP 46 mg/L, Creatinine1.3 mg/dL. Wound culture yielded *Klebsiella* spp. which was sensitive to gentamicin.

Findings were consistent with infected full-thickness thermal vaginal necrosis.

### Therapeutic intervention

Empirical antibiotics were initiated as Ceftriaxone 1 g intravenously every 12 h and Metronidazole 500 mg intravenously every 8 h. On day fourth of the treatment, antibiotic escalation was performed based on culture results, and gentamicin 80 mg intravenously every 8 h was started. Gentamicin was completed 5 days.

Given extensive circumferential necrosis, high risk of ascending infection, advanced prolapse, advanced patient age, no expectation of future sexual activity.

Definitive surgical management was undertaken.

**Figure 2:**
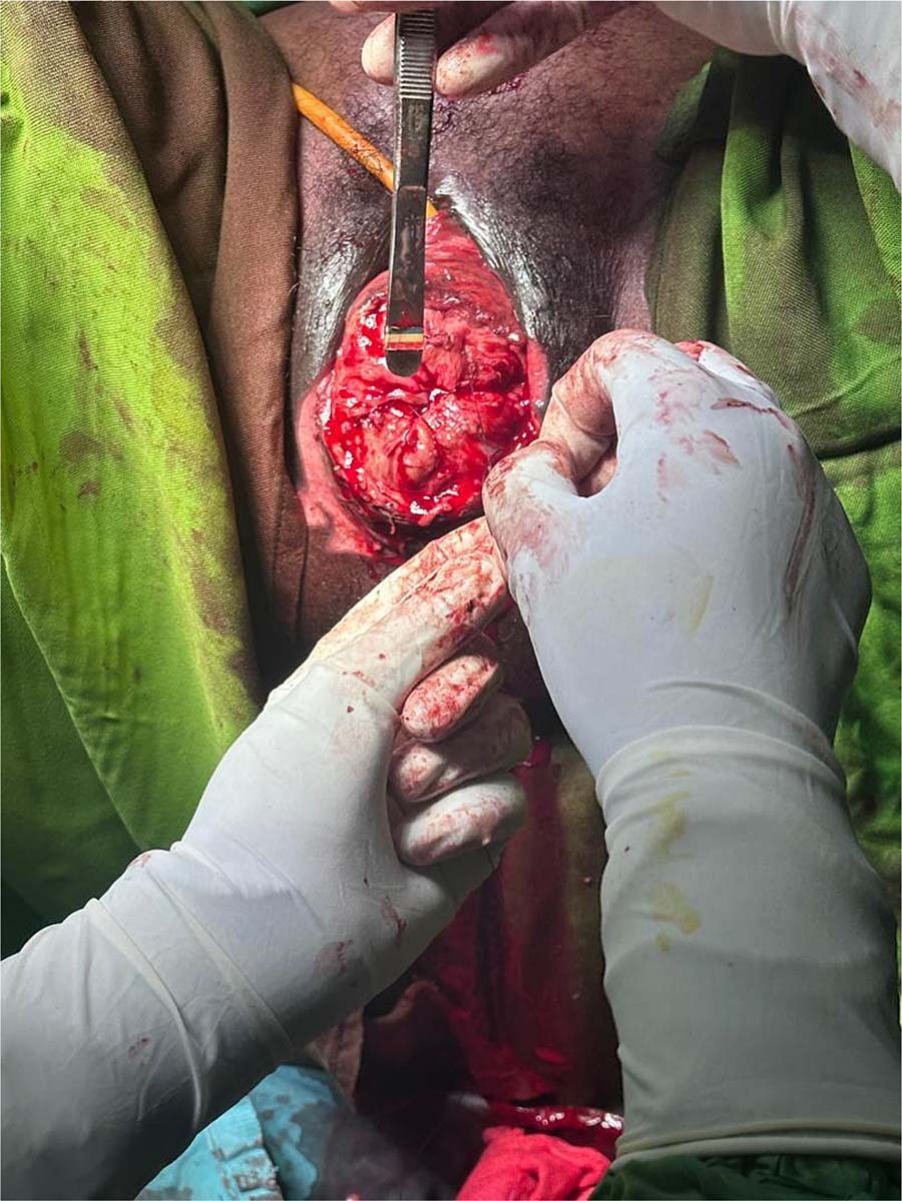
Intraoperative view following extensive debridement showing viable bleeding tissue margins.

**Figure 3:**
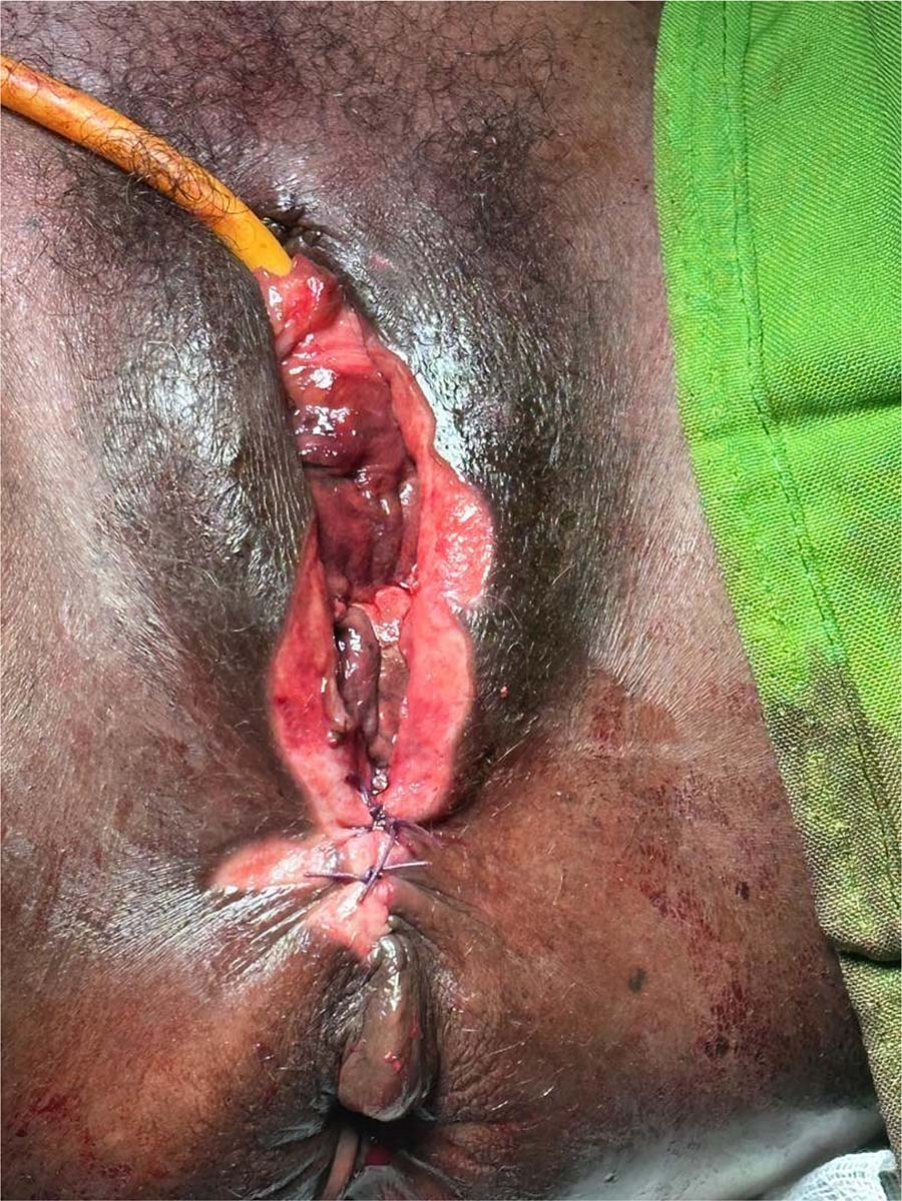
Immediate postoperative appearance after colpocleisis demonstrating obliteration of the vaginal canal.

Intraoperative findings confirmed complete mucosal and submucosal necrosis. Extensive debridement was performed until viable bleeding margins were obtained (Fig. 2). Obliterative surgery with colpocleisis was then carried out to eliminate dead space and restore pelvic support (Fig. 3).

### Follow-up

Postoperative recovery was uneventful resolution of fever, decline in inflammatory markers, restoration of spontaneous urination. The patient was discharged on postoperative day eight.

## Discussion

Thermal injury induces coagulative necrosis via protein denaturation and microvascular occlusion. In genital tissue, sustained heat exposure can lead to progressive ischemia and superinfection, culminating in full-thickness destruction.

The circumferential pattern observed suggests prolonged, uncontrolled thermal application. While genital injury related to harmful traditional practices such as female genital mutilation has been extensively documented [2–4], complete circumferential vaginal wall necrosis requiring obliterative surgery is exceedingly rare.

Colpocleisis is typically indicated for elderly women with advanced pelvic organ prolapse who no longer desire vaginal intercourse [5]. In this case, however, indications extended beyond prolapse management and included massive structural tissue loss, elimination of necrotic dead space, prevention of pelvic sepsis, functional pelvic reconstruction.

Untreated vaginal necrosis may progress to pelvic abscess, rectovaginal fistula, urosepsis, chronic pelvic pain.

From a global health perspective, harmful traditional practices remain preventable causes of severe gynecologic morbidity. The World Health Organization has emphasized the importance of culturally sensitive educational interventions to eliminate such practices [1, 2].

This case highlights the need for clinician awareness in regions where traditional cauterization persists and underscores the potential requirement for radical reconstructive surgery.

## Conclusion

Traditional vaginal cauterization can result in catastrophic full-thickness vaginal necrosis necessitating extensive surgical intervention. Early diagnosis, aggressive infection control, and individualized surgical planning are essential to prevent systemic complications.

Preventive public health strategies targeting harmful traditional practices are urgently required.
